# Improving Lime-Based Restoration Mortars: Effect of Type and Utilization Rate of Binder and Aggregate

**DOI:** 10.3390/ma18050961

**Published:** 2025-02-21

**Authors:** Tuğçe İsafça-Kaya, Yahya Kaya, Naz Mardani, Ali Mardani, Adem Doğangün

**Affiliations:** 1 Department of Civil Engineering, Faculty of Engineering, Bursa Uludag University, Bursa 16059, Turkey; tugcekaya@uludag.edu.tr (T.İ.-K.); 512126007@ogr.uludag.edu.tr (Y.K.); alimardani@uludag.edu.tr (A.M.); 2 Department of Mathematics Education, Bursa Uludag University, Bursa 16059, Turkey; nazmardani@uludag.edu.tr

**Keywords:** lime-based repair mortar, Khorasan mortar, metakaolin, blast furnace slag, mechanical properties

## Abstract

Lime-based mortars, frequently used in historic structures, are classified as hydraulic and non-hydraulic according to how they gain strength. In the past, various methods were used to improve the strength and durability properties of lime-based hydraulic mortars such as Khorasan mortar. Today, in studies carried out to increase the strength of lime-based mortars, the effects of binders, aggregates, and additives, which are the basic components of the mortar, are examined. In this study, the mechanical properties of Khorasan mortar mixtures, which are frequently used in the restoration of historical structures, were examined under the influence of different parameters. In particular, the effects of variables such as aggregate type and ratio (river sand and crushed brick), binder type and ratio (natural hydraulic lime, metakaolin, and blast furnace slag), and water/total dry material ratio on the strength of mortars were investigated experimentally. In the experimental study, two different aggregate types (river sand and crushed brick) were used in 1/3 and 1/2 ratios, and three different binders (natural hydraulic lime, metakaolin, and blast furnace slag) were used in different ratios. The water-to-total-dry-material ratios were set at 0.2 and 0.25. Standard test samples were then created from the prepared mortar mixtures, and their flexural and compressive strengths were assessed at 28 and 56 days. A statistical analysis of the experimental data was conducted using the Taguchi method, allowing for a detailed examination of how the different parameters influenced the strength of the mortars. Through this analysis, the optimal mixture ratios that maximized mortar strength were successfully identified.

## 1. Introduction

Lime mortars have a deep-rooted history and have been widely used in the construction of structures from ancient times to the 21st century. Numerous historical sites and research records indicate that lime was widely used in ancient buildings [1]. During various studies, it was determined that ancient examples dating back to 12,000 BC were encountered in regions in Türkiye and Palestine [2,3,4]. Lime–pozzolana mortars, known for their high strength and hardening properties underwater, were widely used in Roman-era structures [5].

**Figure 1:**
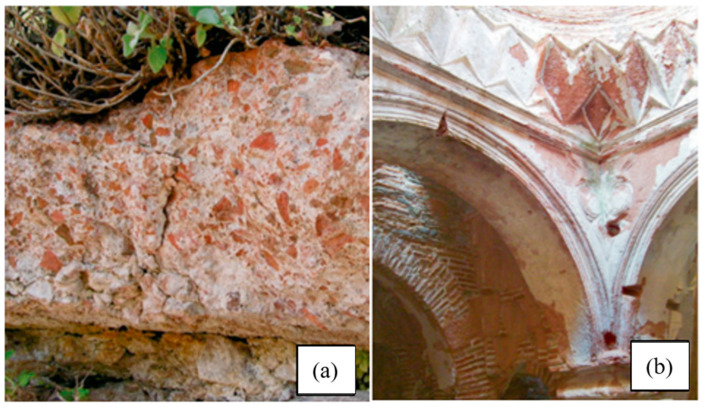
Khorasan mortar used as a floor covering in the Aigai archaeological site (**a**); Khorasan plaster used in the transition elements of an Ottoman bath in Türkiye (**b**) [9].

Khorasan mortar, found in historical structures during the Ottoman, Seljuk, Byzantine, and Roman periods, is a lime-based hydraulic masonry/plaster mortar. It is distinguished from other lime-based mortars due to the crushed brick/brick dust it contains. It is also called cocciopesto and homra in different cultures. Due to its hydraulic properties, this material was often chosen for structures in contact with water, particularly during the Ottoman period [6,7,8]. Some examples of Khorasan mortar used in buildings in the past are given in Figure 1.

Throughout history, efforts have been made to enhance the strength and durability of lime-based mortars. Various methods have been employed to improve these properties, considering the technological possibilities of each period. The inclusion of pozzolan and fibers in the mixtures was intended to enhance the strength of the mortar, while the addition of various organic additives aimed to improve its durability [10,11,12,13,14].

The choice of materials for repairing historically significant structures, which are important for cultural heritage, must be made with great care. The material to be preferred should be physically, chemically, and structurally compatible with the original structure [8,15]. In the past, as a result of ignoring this approach, restoration works using repair materials that were not compatible with the original structure achieved poor results [8]. For this reason, research on the development of restoration materials that are both compatible with the structure and have improved strength and durability properties is important.

When examining studies focused on enhancing the strength and durability of lime-based repair mortars, it has been found that the impact of various factors is considered. These factors include the use of fibers and chemical and mineral admixtures, as well as the type and ratio of binders and aggregates that make up the essential components of the mortar [16,17,18,19].

Aggregates are one of the basic components of mortar mixtures and are also known as the skeleton of the mortar [20,21]. Therefore, the effect of the main properties of the aggregate such as gradation, type, morphology, and pore structure on the mechanical and durability performance of the mortar was examined [22]. Research studies have demonstrated that when silica-containing aggregates are used in mixtures, calcium silicate hydrate (C-S-H) crystals form within the aggregate grains and cracks over time, contributing to the overall strength. Additionally, using aggregates with the optimum size distribution and morphology positively impacts the porosity and permeability of the mortar, ultimately improving its mechanical properties [15,23,24,25,26].

Another basic component of mortar mixtures is the binder. Many studies and field research on historical buildings have shown that lime is a widely used building material. Lime can be divided into two groups according to the way it gains strength: air lime and hydraulic lime. Natural hydraulic lime (NHL) is a binder type different from hydrated lime and is used in cement mixtures that harden by reacting with water due to the siliceous material content in limestone [27]. However, lime mortars have disadvantages compared to cement-based mortars due to their slow strength gain and low final strength [28]. For this reason, the use of pozzolan is frequently preferred in lime-based mortars.

Pozzolans are examined in two main groups according to their origins, natural and artificial. Artificial pozzolans are inorganic materials, mostly containing amorphous silica and alumina, usually obtained as by-products of industrial processes. In this group, materials such as fly ash, blast furnace slag, silica fume, and metakaolin are the main examples [29]. While air lime-based mortars gain hydraulic binding properties with the addition of pozzolan, there are also studies in the literature showing that the strength values of hydraulic lime-based mortars increase [28,30,31,32].

As seen in studies, attempts were made to develop lime-based mortars within the technological possibilities of each period. The use of pozzolanic material and fiber and the addition of various additives to the mixtures can be mentioned as examples of this situation. In this context, research on the types and ratios of materials forming lime-based mortar mixtures is frequently encountered in studies conducted today (Table 1).

When studies on lime-based mortars are examined, it is seen that studies aimed at improving the mechanical and durability properties of these materials are gaining importance. However, it is noteworthy that studies carried out on Khorasan mortar, one of the traditional lime-based mortar types in similar areas, are relatively more limited. This study aims to provide a unique perspective on the engineering properties of these materials by comprehensively evaluating the mechanical performances of Khorasan mortar samples with 24 different mixing ratios determined at different curing times. The parameters affecting the flexural and compressive strengths of the samples include aggregate type and ratio (river sand and crushed brick), binder type and ratio (natural hydraulic lime, metakaolin, and blast furnace slag), water/total dry material ratio, and curing time, and the effects of these variables on the mechanical properties are analyzed in detail. The obtained data are evaluated with the Taguchi method, which offers a systematic approach in experimental design, and the most effective parameters are determined statistically. This study aims to contribute to the field of restoration of historical buildings and sustainable building materials by deeply examining the effects of Khorasan mortar components on engineering performance.

## 2. Materials and Methods

**Figure 2:**
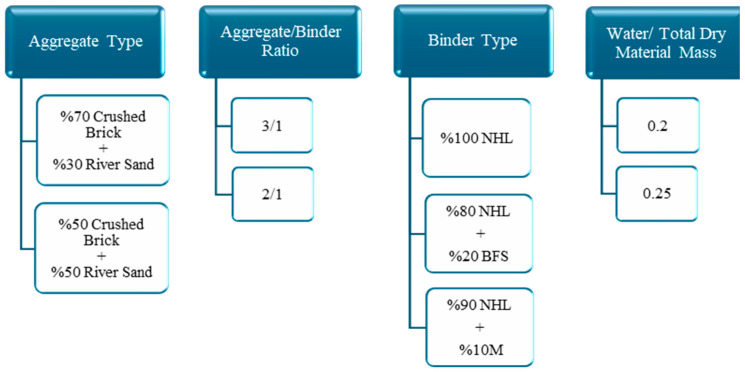
Main and sub-variables taken into consideration when creating mortar mixtures.

Within the scope of this study, the effects of different parameters on the flexural and compressive strengths of Khorasan mortar samples were investigated comprehensively. As shown in detail in Figure 2, these parameters consist of nine variables grouped under four main headings. This study first started with the preparation of mortar mixtures; at this stage, 24 different mortar mixtures were produced using variables such as aggregate type and ratio (river sand and crushed brick), binder type and ratio (natural hydraulic lime, metakaolin, and blast furnace slag) and water/total dry material ratio. The prepared samples were subjected to strength tests after 28 and 56 days of curing and the obtained data were recorded. The obtained results were analyzed using the Taguchi method for statistical evaluation. Detailed information on the properties of the materials used in this study, the preparation process of the mortar mixtures, and the applied experimental methods is presented below.

### 2.1. Materials

#### 2.1.1. Binding Materials

The Khorasan mortar prepared within the scope of this study is lime-based. It is known in the literature to be produced using different types of lime. In this study, NHL 3.5 class natural hydraulic lime (Tekno Construction Chemicals, Istanbul, Türkiye), which complies with the EN 459-1 [43] standard, was preferred due to its high performance in terms of mechanical properties. According to the EN 459-2 [44] standard, the 28-day flexural and compressive strengths of the lime used were determined as 2.22 MPa and 4.89 MPa, respectively.

Metakaolin (M) and blast furnace slag (BFS) (Nuh Cement, Gebze, Türkiye) used as pozzolan were obtained from industrial production companies. Information on the physical and chemical properties of the natural hydraulic lime and pozzolans used within the scope of this study is given in Table 2.

#### 2.1.2. Aggregate

In this study, crushed brick (CB) and standard river sand (RS) were used as aggregates. CB was produced by crushing and grinding blended brick using a jaw crusher. All aggregates were used by sifting through a 2 mm sieve. According to EN 1097-6 Standard [45], the saturated surface dry specific gravities of crushed brick and river sand were measured as 2.18 and 2.60, and the water absorption capacity was measured as 15.11% and 1.31%, respectively.

#### 2.1.3. Fiber

All mixtures prepared within the scope of this study contain 3 mm long polypropylene fibers. Fibers was used at a volume ratio of 0.04%. This optimal amount was determined through preliminary tests conducted earlier [46].

### 2.2. Preparation of Mixtures

Mortar mixtures prepared within the scope of this study were formed by changing the aggregate content (aggregate type and amount), binder content (binder type and amount), binder/aggregate ratio, and water/total dry material mass ratios. Accordingly, the aggregate composition was determined with two different ratios 70% crushed brick–30% river sand and 50% crushed brick–50% river sand. The binder content was prepared with three different variations 100% natural hydraulic lime, 90% natural hydraulic lime–10% metakaolin, and 80% natural hydraulic lime–20% blast furnace slag. In addition, the water/total dry material ratio was applied at two levels, 0.2 and 0.25.

A total of 24 mortar mixtures were prepared by considering the variables determined within the scope of this study. The mixtures were named based on their aggregate content (70CB and 50CB), binder content (NHL, M, BFS), aggregate/binder ratio (3, 2), and water/total dry material ratio (0.2, 0.25). The mixing ratios of the prepared mortar mixtures are presented in detail in Table 3.

### 2.3. Test Method

**Figure 3:**
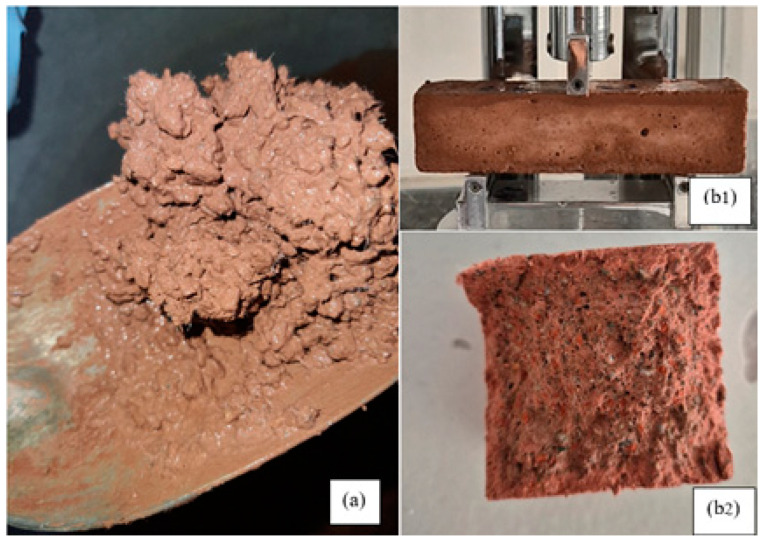
Mortar mixtures prepared within the scope of this study: (**a**) fresh state (**b1,b2**) hardened state.

Mortar mixtures were prepared in accordance with the ASTM C109 standard [47] and cast into 40 × 40 × 160 mm prismatic molds. The samples were initially cured in the molds for two days under controlled conditions at a temperature of 20 ± 2 °C and a relative humidity of 65 ± 5%. Following this initial curing period, the specimens were demolded and subsequently transferred to lime-saturated water, where they were stored until the designated testing day. The flexural and compressive strengths of the samples were evaluated at 28 and 56 days in accordance with the EN 196-1 standard [48], as illustrated in Figure 3.

### 2.4. Experimental Design with Taguchi Method

In this study, ANOVA and Taguchi methods were employed to determine the optimal mix design for Khorasan mortar mixtures. In the Taguchi method, signal-to-noise (S/N) ratios were calculated using the “nominal is best”, “larger is better”, and “smaller is better” approaches, depending on the type of characteristic [49]. In this study, the “largest is the best” approach proposed by Mandal et al. [50] was used as the objective function, as shown in Equation (1).

(1) $$\frac{S}{N}=-10\log\;(\frac{1}{n}\sum_{i=1}^{n}1/{yi}^{2})$$

Here, *y_i_* is the data observed in the *i*th experiment and *n* is the number of observations made in the experiment.

In this study, the control factors were selected as curing time, water/dry material mass ratio, aggregate/binder ratio, aggregate type, and binder type. The levels of the selected factors are shown in Table 4. The most suitable orthogonal array was selected as L36(2^4 3^1) to determine the optimum mixture design for maximum strength performance and to analyze the effects of the parameters. The L36 mixed orthogonal array was used to analyze the experimental results, as shown in Table 5.

## 3. Results and Discussion

### 3.1. Flexural and Compressive Strength

**Figure 4:**
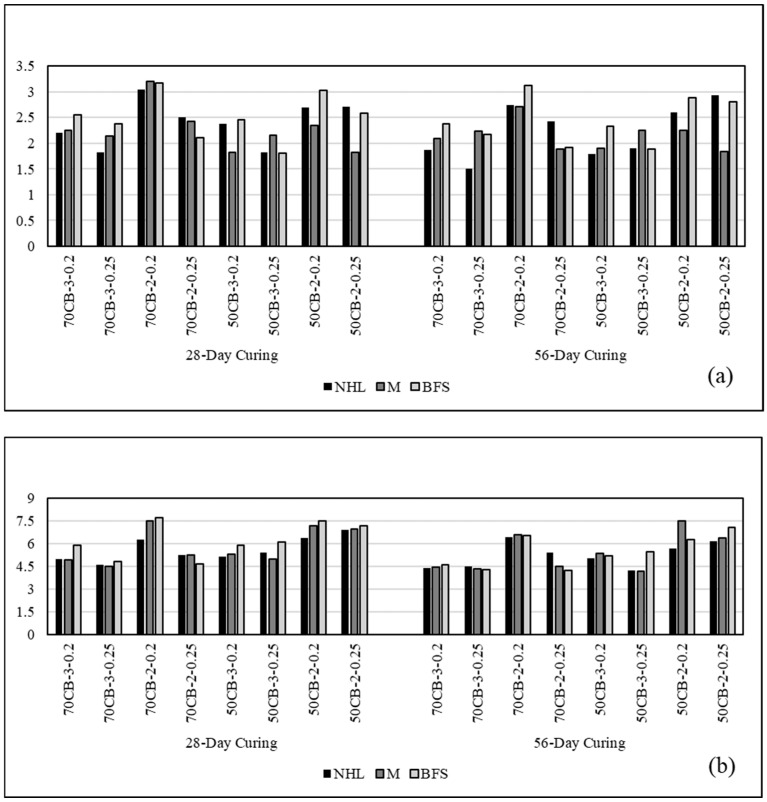
Flexural (**a**) and compressive (**b**) strength values of mortar specimens at 28 and 56 days. The full names of the abbreviations listed in the figure are as follows: natural hydraulic lime (NHL), metakaolin (M), blast furnace slag (BFS), crushed brick (CB), river sand (RS).

The flexural and compressive strength values of the mortar mixture samples prepared for this study are presented in Table 6 and shown comparatively in Figure 4.

As can be seen from the table, the strength performance was generally negatively affected by the increase in the curing time, regardless of the binder and aggregate type. This situation became more evident with the substitution of metakaolin (M) and blast furnace slag (BFS).

The mortars produced for this study have hydraulic properties, meaning they are designed to gain strength when in contact with water. It is expected that this situation will become evident with the substitution of pozzolan in some of the mixtures. However, it is known that carbonation occurs due to the carbon dioxide penetrating the hydraulic lime mortars after they are set [51]. Since it is not possible for samples completely cured in water to access CO_2_, it was suggested by some researchers that non-carbonated lime in mortar mixtures may reduce the strength of the mortar [52].

When the effect of binder type on strength is examined, the strength is positively affected by the use of metakaolin and blast furnace slag. It was determined that BFS increases the strength even more compared to metakaolin. As emphasized before, natural hydraulic lime (NHL) gains strength as a result of the reaction of C_2_S in its structure with water [51]. Substituted pozzolans with varying chemical compositions can facilitate the pozzolanic reaction with natural hydraulic lime, resulting in the formation of calcium silicate hydrate (CSH) from calcium hydroxide (Ca(OH)_2_). In the mixtures prepared within the scope of this study, this situation is especially prominent with the use of metakaolin. Metakaolin, being very fine and with its high reactive silica content, shows a pozzolanic reaction with Ca(OH)_2_ and causes higher strength performance compared to mixtures containing only NHL. Another pozzolan used in this study is BFS. BFS is known for having properties that are most similar to those of cement due to its calcium oxide (CaO) content, which contrasts with that of metakaolin. As a result, the pozzolanic effect of BFS primarily stems from its silicate content. This characteristic accounts for the superior strength performance of BFS.

In the study conducted by Grilo et al. [30], the strength values of lime-based mortars containing metakaolin were examined under different curing conditions. As a result of the study, it was determined that the strength values of mortars containing metakaolin decreased at a higher rate than those without metakaolin. It was suggested that this situation was due to the unstable compounds formed as a result of the pozzolanic reaction between metakaolin and lime. Vavricuk et al. [28] examined how the fineness and substitution rate of metakaolin affect the properties of lime-based mortars in both fresh and hardened states. As a result of the study, it was observed that the use of metakaolin had a positive effect on the hardened properties of the mortars. In addition, it was determined that the compounds formed as a result of the reaction with lime differed depending on the fineness value of the metakaolin used.

There are studies in the literature indicating that BFS is used as an aggregate or binder in lime-based mortars and that it improves the strength and durability properties of the mortar [53,54,55]. In the study conducted by Shang et al. [56], it was determined that the BFS ratio added to NHL mortars increased the compressive and flexural strength of the mortars up to a certain point. The increase in the amount of C-S-H gel formed as a result of the pozzolanic reactions of BFS improved the mechanical properties of the mortars. However, it was emphasized that excessive BFS addition caused a decrease in strength due to the lack of sufficient calcium hydroxide for the reactions.

The strength and workability of lime mortars are closely related to the interaction between the binder and the aggregate. While the type of binder and particle size distribution determines the strength gain mechanisms of the mortar and hence its strength, the mineralogical properties and size distribution of the aggregates affect the physical properties of the mortar [57,58]. It is known that the effect of the aggregate/binder ratio on the mortar’s fresh and hardened properties varies depending on the type of lime used as a binder. Apostolopoulou et al. [59] conducted their research utilizing NHL 2 and NHL 3.5 lime as binders, combined with a specific type of river sand as the aggregate. They determined that the optimum binder/aggregate ratio in terms of strength for lime containing NHL 2 was lower than for mixtures containing NHL 3.5.

**Figure 5:**
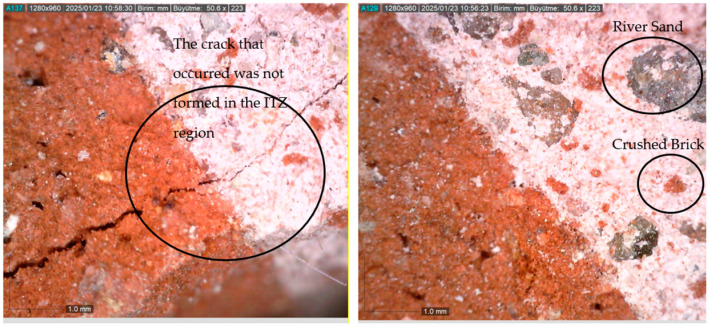
Optical microscope images of mortar–crushed brick adhesion.

Examining the effect of the aggregate content in the mixtures, it was found that reducing the amount of crushed brick in the aggregate from 70% to 50% positively affected the strength performance in all mixtures. It is known that crushed brick contains more voids compared to river sand aggregate and has lower strength but consists of more angular grains. As seen in Figure 5, the ITZ formed around the crushed brick is stronger. This situation is also because the crushed brick aggregate has a more angular and rough structure than the river sand aggregate. In the studies of Lanas et al. [15] and Arizzi and Cultrone [60], the important role of the shape of the aggregates on the strength of the mortars was revealed. It was emphasized that rounded aggregates reduce the strength by creating larger pores in the mortars, while angular and rough aggregates increase the strength by providing a better interaction with the binder.

In the mortar mixtures prepared within the scope of this study, the effect of river sand and crushed brick content may vary according to the strength performance of the dough phase. When crushed brick is used in high-strength matrices, fractures occur in the aggregate phase due to the weakness of the aggregate phase. However, in low-strength mixtures, the fact that the aggregate phase has strength close to that of the dough phase eliminates this situation. In this case, it is thought that the use of stronger aggregates positively affects the strength. In Khorasan mortar, the strength-reducing effect of crushed brick is less prominent due to the low strength of the dough phase.

When traditional Khorasan mortar mixtures are examined, it is seen that lime–brick dust/crushed brick mixtures are used especially in humid regions. It is known that brick aggregates containing amorphous clay minerals react with lime binder and form additional bonds in mortars and plasters, and thus the mechanical properties of the mortar are improved [61,62]. Since aggregates in the 0–2 mm sieve range were used within the scope of this study, the aggregate roughness strengthens the ITZ, as well as increasing cohesiveness and entraining air into the mixture. Therefore, it is thought that the low strength of mixtures with high crushed brick content is due to this.

When the effect of the aggregate/binder ratio on strength was examined, the strength was positively affected by decreasing the ratio from 3 to 2 in all mixtures. It is believed that this situation arises from an increase in the aggregate phase within the mixture, alongside the paste phase’s insufficient volume to adequately cover the aggregates [54]. In lime-based mortar systems, the water/binder ratio is a critical parameter in terms of its effects on the mechanical properties and longevity of mortars. Studies in the literature reveal the direct relationship between this ratio and hydration kinetics, porosity, and microstructure. For example, in a study conducted by Xu et al. [63], the effect of the water/binder ratio on the hydration reaction was examined and it was stated that hydration accelerated in a certain range. Papayianni and Stefanidou [34] emphasized that the water/binder ratio is the most important factor that directly affects the porosity structure of mortars.

In the mortar mixtures within the scope of this study, the water/dry material mass ratio was used instead of the water/binder ratio. In this case, as expected, the results obtained showed that the durability performance was negatively affected by the increase in water content.

To evaluate the adhesion performance of the mixture with the highest strength performance with the crushed brick, images were taken with an optical microscope. The images obtained are given in Figure 5. As seen in the figure, it is seen that the mortar mixture exhibits strong adhesion performance with the crushed brick. When the load is applied to bricks covered with filling mortar, the fact that there is no fracture at the brick–mortar interface reveals that they are bonded homogeneously.

### 3.2. Evaluation of Analysis Results

#### 3.2.1. Analysis of Signal-to-Noise (S/N) Ratio

The S / N ratios and means values for compressive strength parameters are shown in Table 7.

**Figure 6:**
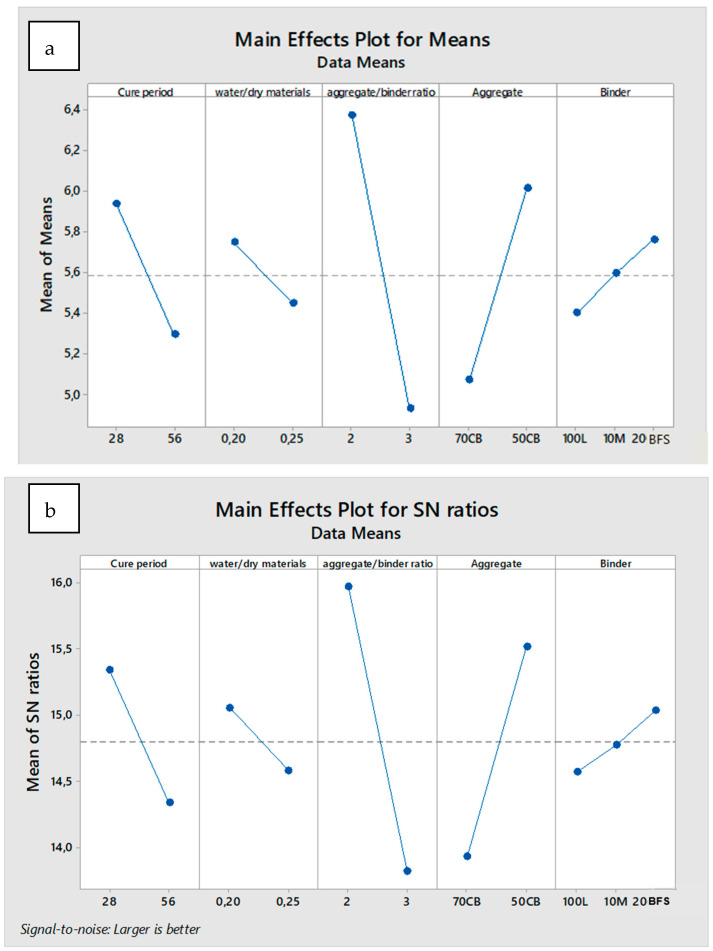
Influence levels and significance graph of effective parameters for compressive strength (**a**) according to S/N ratio and (**b**) according to mean values. The full names of the abbreviations listed in the figures are as follows: natural hydraulic lime (L), metakaolin (M), blast furnace slag (BFS), crushed brick (CB).

This table, prepared using the Taguchi technique, shows the control factor levels of optimum mix design for maximum compressive strength. The level values of control factors given in Table 8 are shown graphically in Figure 6. The optimum parameters of control factors for maximizing compressive strength can be easily determined from these graphs. The best level for each control factor was found according to the highest S/N ratio and the highest mean value at the level of that control factor. Accordingly, the different mix designs providing the highest compressive strength were determined as Level 1, S/N = 5.34 for factor A, Level 1, S/N = 15.05 for factor B, Level 1, S/N = 15.95 for factor C, Level 2, S/N = 15.51 for factor D, and Level 3, S/N = 15.0.3 for factor E. As a result, it was determined that the maximum compressive strength was obtained by curing the mixture with 20% BFS, 50% crushed brick, aggregate/binder ratio of 2, and water/dry material mass ratio of 0.2 for 28 days.

#### 3.2.2. ANOVA Method

**Figure 7:**
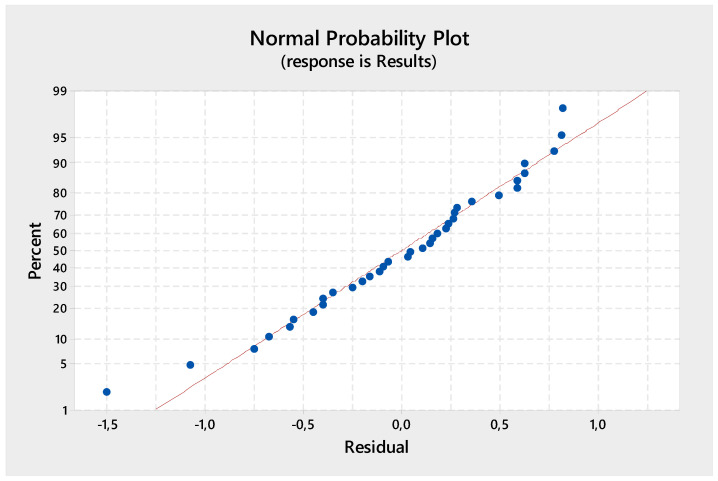
ANOVA model fit.

The results of the ANOVA analysis for compressive strength are presented in Table 9. The analysis was conducted at a 5% significance level with a 95% confidence interval. The significance of the control factors in ANOVA was assessed by comparing the F-values of each factor. Additionally, a *p*-value lower than 0.05 for a given control factor indicates statistical significance. The effect ratio provided in the last column of Table 8 represents the percentage contribution of each parameter, reflecting its influence on process performance. According to Table 8, the percentage contributions of binder type, aggregate type, aggregate/binder ratio, water-to-dry-material-mass ratio, and curing time to compressive strength were determined as 7.75%, 11.89%, 48.74%, 10.61%, and 17.06%, respectively. These findings indicate that the aggregate/binder ratio has the most significant impact on compressive strength. The error percentage of the ANOVA model was calculated as 3.95%. The accuracy curve of anova is given in Figure 7.

#### 3.2.3. Regression Analysis

Regression analysis is used for modeling and analyzing many variables when there is a relationship between a dependent variable and one or more independent variables [64]. In this study, the dependent variables were selected as compressive strength, while the independent variables were selected as binder type, aggregate type, aggregate/binder ratio, water/dry material mass ratio, and curing time. Regression analysis was used to obtain equations that could estimate the compressive strength of the prepared mixtures. In addition, regression equations were created separately for each aggregate type and binder type. Therefore, the equation of whichever aggregate type and binder type distribution is selected should be used. These estimation equations were made for both linear and quadratic regression models. The estimation equations obtained with the linear regression model of compressive strength are given below (Table 10).

**Figure 8:**
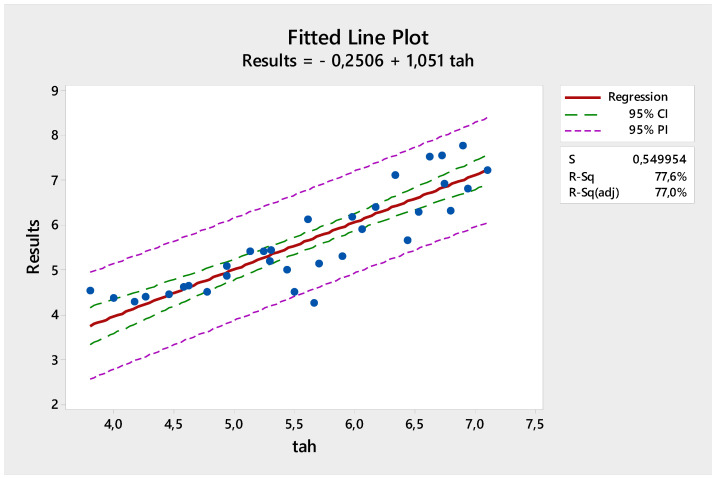
Comparison of linear regression model with experimental results on compressive strength.

Figure 8 shows the comparison of the actual test results obtained with the linear regression model and the predicted values. The R^2^ value of the equation obtained with the linear regression model of compressive strength was found to be 77.17%.

The estimation equation for the second-order regression of compressive strength is given below.

The regression equation is

Results = 8.673 − 2.262 Mean + 0.2984 Mean^2

**Figure 9:**
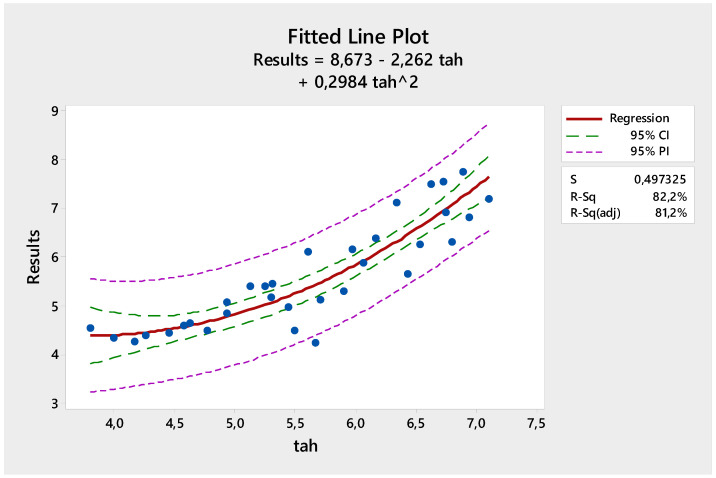
Comparison of second-order regression model with experimental results on compressive strength.

Figure 9 shows the comparison of the test results and the estimated values obtained with the second-order regression model. As can be seen from the figure, there is a very good relationship between the estimated values and the test results. The R^2^ value of the equation obtained with the second-order regression model for compressive strength was calculated as 81.24%. Therefore, more intensive estimated values were obtained with the second-order regression model compared to the linear regression model. As a result, it was shown that the second-order regression model was successful in estimating compressive strength.

#### 3.2.4. Estimation of Optimum Compressive Strength by Taguchi Method, Linear Regression Equations, and Quadratic Regression Equation and Comparison with Experimental Result

For the Taguchi method and regression equations, verification tests of control factors were performed at optimum and random levels. The results obtained are shown in Table 11. It is also understood from the results that the estimated values and experimental data are quite close. It was reported by Kıvak [65] that the error values should be less than 20% for reliable statistical analyses. In this direction, it was understood that the obtained data were at an acceptable level. Therefore, the results obtained from the verification tests reflect successful optimization.

## 4. Conclusions

The results obtained in this study, in which the effects of using different types and ratios of aggregate and pozzolan and two different water/total dry material mass and aggregate/binder ratios on the compressive and flexural strength of lime-based repair mortars were examined, are summarized below:

- Regardless of the binder and aggregate type, the strength performance of the Khorasan mortar was generally negatively affected by the increase in the curing time.
- When the effect of binder type on strength was examined, strength was positively affected by the use of metakaolin and BFS. It was determined that BFS increased strength even more compared to metakaolin.
- The decrease in the ratio of crushed brick aggregate used in the mixtures positively affected the strength performance.
- When the effect of the aggregate/binder ratio on strength was examined, strength was positively affected by a decrease in this ratio from 3 to 2 in all mixtures.
- An increase in the water/total dry material mass ratio negatively affected the strength performance of the mortar samples.
- The optimum levels of the control factors on the strength values were determined using S/N ratios. The parameter that affects the strength the most was determined as the aggregate/binder ratio.
- When the predictions were compared with the experimental results, it was found that the error margin was acceptable.

This study experimentally investigated various parameters influencing the mechanical strength of traditional Khorasan mortar. Specifically, the effects of different binder and aggregate types, aggregate/binder ratios, and water/dry material ratios on mortar strength were evaluated. The findings indicate that extended curing time negatively impacts strength performance, regardless of binder and aggregate type. However, the use of metakaolin and blast furnace slag (BFS) as binders enhanced mechanical strength, with BFS demonstrating superior performance compared to metakaolin. Additionally, optimizing the aggregate/binder ratio and reducing the proportion of crushed brick aggregate positively influenced strength characteristics.

These results provide valuable insights into the performance of Khorasan mortar in restoration and reinforcement applications, aiding in the determination of optimal strength levels. Given that lime-based repair mortars often exhibit low mechanical performance, the incorporation of pozzolanic materials and optimized mixture proportions can enhance their durability. However, it remains essential to preserve the structural authenticity of historical constructions when selecting materials for restoration projects.

Future research should further examine the strength development mechanisms of Khorasan mortar and focus on its adhesion performance to brick substrates through microstructural analysis.

## Figures and Tables

**Table 1:** Some studies about enhancing lime-based mortars.

Lime + Pozzolan	Aggregate	Highlights	Ref.
Hydrated lime, Czech clay shale, metakaolin	Natural quartz and basalt sand, 0–4 mm	Increasing the fineness values of pozzolans has a positive effect on the mortar’s porosity, compressive, and flexural strengths.	[33]
Hydrated lime and natural pozzolan from the island of Milos	River aggregate (fine sand: 0–2 mm/coarse aggregate: 0–4 mm)	Water/binder ratio is the most important parameter affecting porosity. The porosity of lime–pozzolan mortars decreases over time and is strongly affected by curing conditions.	[34]
Natural hydraulic lime, silica fume, blast furnace slag, metakaolin, and brick dust	CEN standard sand, Guiting dust	Using different types of pozzolans in certain proportions, separately and together, increases sensitivity to curing conditions and compressive strength. Curing conditions have a significant effect on the strength of the samples.	[35]
Hydrated lime, metakaolin, and zeolite	CEN standard sand	Metakaolin and zeolite substitution positively affected some strength and durability properties of lime mortars. The products formed because of pozzolanic reactions differed depending on the pozzolan used and the curing medium.	[36]
Air lime and metakaolin	(20 ± 5) °C, (95 ± 5)% RH	Flexural strength decreases as the binder/aggregate ratio of mortars decreases. Mortars with binder/aggregate ratio (1:3) show faster carbonation, which depletes portlandite and inhibits short-term pozzolanic reactions. Conversely, mortars with binder/aggregate ratio (1:1) experience slower carbonation, allowing more lime for longer-term pozzolanic reactions.	[37]
Hydrated lime and natural hydraulic lime	Two types of fine aggregates containing quartz from different sources	Adding hydraulic lime to air lime mortars strengthened the mechanical properties of the mortars while also accelerating the hardening process. A low binder/aggregate ratio results in less dense packing and increased porosity in the mortar microstructure.	[38]
Three types of Korean natural hydraulic lime, silica fume, and blast furnace slag	Sand	With the addition of BFS and SF, physical properties such as compressive strength and setting time could be improved. The fact that SF has very fine particles accelerated its initial hydration and contributed to its strength as a filler.	[39]
Two types of aerial lime, blast furnace slag, and fly ash	River sand	With BFS and FA substitution, the strength gain rate increased, water requirement decreased, and mechanical properties improved. Lime–BFS-based mortars performed better. Lime–FA-based mortars are more advantageous in terms of drying shrinkage resistance.	[40]
Hydrated lime, natural hydraulic lime, and %50 Hydrated lime + %50 Portland cement	Expanded perlite (EP), expanded glass (EG), and natural zeolite	The inclusion of EP and EG in the mortar components significantly increased the thermal insulation properties of the materials and strengthened their resistance to salt effects.	[41]
Natural hydraulic lime, tuff, and metakaolin-expanded perlite	River sand: coarse aggregate (0–8 mm)/fine aggregate (0–4 mm): 2/1	Water-cured samples exhibited better performance in terms of physical and mechanical properties than air-cured samples, regardless of the type of mineral additive.	[42]

**Table 2:** Some physical and chemical properties of NHL, M, and BFS.

Item	Unit	NHL	M	BFS
SiO_2_	%	8.47	56.10	40.17
Al_2_O_3_	%	3.46	40.23	13.81
Fe_2_O_3_	%	0.39	0.85	0.94
CaO	%	53.84	0.19	30.12
MgO	%	-	0.16	6.60
SO_3_	%	-	-	1.73
Na_2_O	%	0.16	0.24	0.52
K_2_O	%	0.27	0.51	0.96
LOI	%	30.41	1.10	0.32
Specific gravity		2.79	2.52	2.90
Specific surface area	cm^2^/g	5850	146,000	4880

The full names of the abbreviations listed in the table are as follows: natural hydraulic lime (NHL), metakaolin (M), blast furnace slag (BFS).

**Table 3:** Amounts of materials used in mixtures.

A	B	A/B	W/Dm	Mixtures	CB (%) *	RS (%) *	NHL (%) **	M (%) **	BFS (%) **
70CB	NHL	3	0.2	70CB-NHL-3-0.2	70	30	100		
		3	0.25	70CB-NHL-3-0.25	70	30	100		
		2	0.2	70CB-NHL-2-0.2	70	30	100		
		2	0.25	70CB-NHL-2-0.25	70	30	100		
	M	3	0.2	70CB-M-3-0.2	70	30	90	10	
		3	0.25	70CB-M-3-0.25	70	30	90	10	
		2	0.2	70CB-M-2-0.2	70	30	90	10	
		2	0.25	70CB-M-2-0.25	70	30	90	10	
	BFS	3	0.2	70CB-BFS-3-0.2	70	30	80		20
		3	0.25	70CB-BFS-3-0.25	70	30	80		20
		2	0.2	70CB-BFS-2-0.2	70	30	80		20
		2	0.25	70CB-BFS-2-0.25	70	30	80		20
50CB	NHL	3	0.2	50CB-NHL-3-0.2	50	50	100		
		3	0.25	50CB-NHL-3-0.25	50	50	100		
		2	0.2	50CB-NHL-2-0.2	50	50	100		
		2	0.25	50CB-NHL-2-0.25	50	50	100		
	M	3	0.2	50CB-M-3-0.2	50	50	90	10	
		3	0.25	50CB-M-3-0.25	50	50	90	10	
		2	0.2	50CB-M-2-0.2	50	50	90	10	
		2	0.25	50CB-M-2-0.25	50	50	90	10	
	BFS	3	0.2	50CB-BFS-3-0.2	50	50	80		20
		3	0.25	50CB-BFS-3-0.25	50	50	80		20
		2	0.2	50CB-BFS-2-0.2	50	50	80		20
		2	0.25	50CB-BFS-2-0.25	50	50	80		20

The full names of the abbreviations listed in the table are as follows: natural hydraulic lime (NHL), metakaolin (M), blast furnace slag (BFS), crushed brick (CB), river sand (RS), water/total dry mass ratio (W/Dm), aggregate/binder ratio (A/B). * ratio by aggregate mass. ** ratio by binder mass.

**Table 4:** Factor parameters and levels.

Parameters	Symbol	Level 1	Level 2	Level 3
Cure Period	A	28	56	
Water/Dry Materials	B	0.2	0.25	
Aggregate/Binder Ratio	C	2	3	
Aggregate	D	70CB	50CB	
Binder	E	NHL	M	BFS

The full names of the abbreviations listed in the table are as follows: natural hydraulic lime (NHL), metakaolin (M), blast furnace slag (BFS), crushed brick (CB), river sand (RS).

**Table 5:** Taguchi L36(2^3 3^1) layout.

Experiment No.	Factor A	Factor B	Factor C	Factor D	Factor E
	*Cure Period*	*W/Dm Ratio*	*A/B Ratio*	*Aggregate*	*Binder*
1	28	0.2	2	70CB	NHL
2	28	0.2	2	70CB	M
3	28	0.2	2	70CB	BFS
4	28	0.2	2	70CB	NHL
5	28	0.2	2	70CB	M
6	28	0.2	2	70CB	BFS
7	28	0.2	3	50CB	NHL
8	28	0.2	3	50CB	M
9	28	0.2	3	50CB	BFS
10	28	0.25	2	50CB	NHL
11	28	0.25	2	50CB	M
12	28	0.25	2	50CB	BFS
13	28	0.25	3	70CB	NHL
14	28	0.25	3	70CB	M
15	28	0.25	3	70CB	BFS
16	28	0.25	3	50CB	NHL
17	28	0.25	3	50CB	M
18	28	0.25	3	50CB	BFS
19	56	0.2	3	50CB	NHL
20	56	0.2	3	50CB	M
21	56	0.2	3	50CB	BFS
22	56	0.2	3	70CB	NHL
23	56	0.2	3	70CB	M
24	56	0.2	3	70CB	BFS
25	56	0.2	2	50CB	NHL
26	56	0.2	2	50CB	M
27	56	0.2	2	50CB	BFS
28	56	0.25	3	70CB	NHL
29	56	0.25	3	70CB	M
30	56	0.25	3	70CB	BFS
31	56	0.25	2	50CB	NHL
32	56	0.25	2	50CB	M
33	56	0.25	2	50CB	BFS
34	56	0.25	2	70CB	NHL
35	56	0.25	2	70CB	M
36	56	0.25	2	70CB	BFS

The full names of the abbreviations listed in the table are as follows: natural hydraulic lime (NHL), metakaolin (M), blast furnace slag (BFS), crushed brick (CB), river sand (RS).

**Table 6:** Flexural and compressive strength values of mortar samples.

Samples	28-Day Curing						56-Day Curing					
	Flexural Strength			Compressive Strength			Flexural Strength			Compressive Strength		
	*NHL*	*M*	*BFS*	*NHL*	*M*	*BFS*	*NHL*	*M*	*BFS*	*NHL*	*M*	*BFS*
70CB-3-0.2	2.2	2.25	2.55	4.98	4.95	5.88	1.87	2.1	2.37	4.38	4.42	4.62
70CB-3-0.25	1.82	2.14	2.37	4.59	4.48	4.84	1.5	2.23	2.17	4.52	4.34	4.26
70CB-2-0.2	3.05	3.21	3.17	6.26	7.53	7.74	2.74	2.71	3.12	6.44	6.59	6.53
70CB-2-0.25	2.51	2.42	2.11	5.24	5.26	4.66	2.43	1.89	1.92	5.43	4.48	4.23
50CB-3-0.2	2.38	1.83	2.45	5.12	5.28	5.88	1.79	1.91	2.33	5.05	5.38	5.17
50CB-3-0.25	1.83	2.16	1.80	5.40	4.97	6.09	1.91	2.25	1.88	4.24	4.16	5.48
50CB-2-0.2	2.70	2.35	3.03	6.38	7.20	7.51	2.60	2.25	2.88	5.65	7.50	6.29
50CB-2-0.25	2.71	1.82	2.58	6.90	6.95	7.20	2.94	1.84	2.80	6.15	6.39	7.10

The full names of the abbreviations listed in the table are as follows: natural hydraulic lime (NHL), metakaolin (M), blast furnace slag (BFS), crushed brick (CB), river sand (RS).

**Table 7:** S/N ratios and mean values for compressive strength parameters.

Experiment No	Control Factors					Compressive Strength (Mpa)	S/N Ratio for Compressive Strength	Means for Compressive Strength
	Cure Period	W/Dm	A/B	Aggregate	Binder			
1	28	0.2	2	70CB	NHL	6.26	15.9315	6.26
2	28	0.2	2	70CB	M	7.53	17.5359	7.53
3	28	0.2	2	70CB	BFS	7.74	17.7748	7.74
4	28	0.2	2	70CB	NHL	6.26	15.9315	6.26
5	28	0.2	2	70CB	M	7.53	17.5359	7.53
6	28	0.2	2	70CB	BFS	7.74	17.7748	7.74
7	28	0.2	3	50CB	NHL	5.12	14.1854	5.12
8	28	0.2	3	50CB	M	5.28	14.4527	5.28
9	28	0.2	3	50CB	BFS	5.88	15.3875	5.88
10	28	0.25	2	50CB	NHL	6.90	16.7770	6.90
11	28	0.25	2	50CB	M	6.80	16.6502	6.80
12	28	0.25	2	50CB	BFS	7.20	17.1466	7.20
13	28	0.25	3	70CB	NHL	4.59	13.2363	4.59
14	28	0.25	3	70CB	M	4.48	13.0256	4.48
15	28	0.25	3	70CB	BFS	4.84	13.6969	4.84
16	28	0.25	3	50CB	NHL	5.40	14.6479	5.40
17	28	0.25	3	50CB	M	4.97	13.9271	4.97
18	28	0.25	3	50CB	BFS	6.09	15.6923	6.09
19	56	0.2	3	50CB	NHL	5.05	14.0658	5.05
20	56	0.2	3	50CB	M	5.38	14.6156	5.38
21	56	0.2	3	50CB	BFS	5.17	14.2698	5.17
22	56	0.2	3	70CB	NHL	4.38	12.8295	4.38
23	56	0.2	3	70CB	M	4.42	12.9084	4.42
24	56	0.2	3	70CB	BFS	4.62	13.2928	4.62
25	56	0.2	2	50CB	NHL	5.65	15.0410	5.65
26	56	0.2	2	50CB	M	7.50	17.5012	7.50
27	56	0.2	2	50CB	BFS	6.29	15.9730	6.29
28	56	0.25	3	70CB	NHL	4.52	13.1028	4.52
29	56	0.25	3	70CB	M	4.34	12.7498	4.34
30	56	0.25	3	70CB	BFS	4.26	12.5882	4.26
31	56	0.25	2	50CB	NHL	6.15	15.7775	6.15
32	56	0.25	2	50CB	M	6.39	16.1100	6.39
33	56	0.25	2	50CB	BFS	7.10	17.0252	7.10
34	56	0.25	2	70CB	NHL	5.43	14.6960	5.43
35	56	0.25	2	70CB	M	4.48	13.0256	4.48
36	56	0.25	2	70CB	BFS	4.23	12.5268	4.23

The full names of the abbreviations listed in the table are as follows: natural hydraulic lime (NHL), metakaolin (M), blast furnace slag (BFS), crushed brick (CB), river sand (RS), water/total dry mass ratio (W/Dm), aggregate/binder ratio (A/B), signal-to-noise ratio (S/N ratio).

**Table 8:** S/N and mean response results for compressive strength.

Response Table for Signal-to-Noise Ratios						Response Table for Means					
Level	Cure Period	W/Dm	A/B	Aggregate	Binder	Level	Cure Period	W/Dm	A/B	Aggregate	Binder
1	15.34	15.05	15.97	13.93	14.57	1	5.939	5.751	6.377	5.075	5.405
2	14.34	14.58	13.82	15.51	14.77	2	5.298	5.454	4.933	6.018	5.597
3					15.03	3					5.765
Delta	1	0.47	2.15	1.59	0.46	Delta	0.641	0.297	1.444	0.943	0.361
Rank	3	4	1	2	5	Rank	3	5	1	2	4

The full names of the abbreviations listed in the table are as follows: natural hydraulic lime (NHL), metakaolin (M), blast furnace slag (BFS), crushed brick (CB), river sand (RS), water/total dry mass ratio (W/Dm), aggregate/binder ratio (A/B).

**Table 9:** Effect ranking according to ANOVA results.

Source	Degre of Freedom (DoF)	Sum of Squares (SS)	Mean Square (MS)	F-Value	*p*-Value	Impact Rates (%)
Cure period	1	6.4601	6.4601	18.58	0	17.06%
W/Dm	1	2.576	2.576	7.41	0.011	10.61%
A/B	1	22.3887	22.3887	64.4	0	48.74%
Aggregate	1	3.1625	3.1625	9.1	0.005	11.89%
Binder	2	1.2622	0.6311	1.82	0.181	7.75%
Error	29	10.0826	0.3477			3.95%
Total	35	45.932				100.00%

**Table 10:** Estimation equations obtained with the linear regression model.

Aggregate	Binder			
70CB	100L	Result	=	12.80 − 0.03026 Cure period − 10.70 water/dry materials − 1.577 aggregate/binder ratio
70CB	10M	Result	=	13.08 − 0.03026 Cure period − 10.70 water/dry materials − 1.577 aggregate/binder ratio
70CB	20BFS	Result	=	13.26 − 0.03026 Cure period − 10.70 water/dry materials − 1.577 aggregate/binder ratio
50CB	100L	Result	=	13.39 − 0.03026 Cure period − 10.70 water/dry materials − 1.577 aggregate/binder ratio
50CB	10M	Result	=	13.68 − 0.03026 Cure period − 10.70 water/dry materials − 1.577 aggregate/binder ratio
50CB	20BFS	Result	=	13.85 − 0.03026 Cure period − 10.70 water/dry materials − 1.577 aggregate/binder ratio

**Table 11:** Comparison of regression estimates with experimental results.

	Taguchi Method		Linear Regression		Quadratic Regression	
	28-0.2-3-70CB-100L	56-0.2-3-50CB-100L	28-0.2-3-70CB-100L	56-0.2-3-50CB-100L	28-0.2-3-70CB-100L	56-0.2-3-50CB-100L
Experimental result	4.98	5.38	4.98	5.38	4.98	5.38
Estimation	5.52	5.12	5.65	5.11	5.4	5.21
Error (%)	10.8	4.8	14.2	5	8.4	3.1

## Data Availability

The original contributions presented in this study are included in the article; further inquiries can be directed to the corresponding author.
